# What Do Teachers Know and Should Know About Developmental Language Disorder? Examining Knowledge, Attitudes, and Views of Teachers in Cyprus

**DOI:** 10.3390/children13050663

**Published:** 2026-05-09

**Authors:** Elena Theodorou, Marousa Kyritsi, Rouzana Komesidou

**Affiliations:** 1 Department of Rehabilitation Sciences, Cyprus University of Technology, Limassol 3036, Cyprus; marizakyritisi@gmail.com (M.K.); rouzana.komesidou@cut.ac.cy (R.K.); 2 Mosinian Research & Consultancy, Limassol 3046, Cyprus

**Keywords:** developmental language disorder, teachers, knowledge, professional development

## Abstract

**Highlights:**

**What are the main findings?**

- Teachers demonstrated basic familiarity with Developmental Language Disorder (DLD) but lacked practical competence in accurate identification and effective classroom support.
- Empirical data revealed significant gaps in teachers’ knowledge, attitudes, and roles, which guided the development of the Basic-DLD Guide.
- The findings highlight the need for structured, evidence-based professional development tailored to teachers’ needs regarding DLD.

**What are the implications of the main findings?**

- Trained teachers can significantly contribute to early identification and intervention of DLD, thereby enhancing children’s literacy, academic performance, and social inclusion.
- DLD-specific content should be integrated into pre-service and in-service teacher education. The Basic-DLD Guide can structure training on knowledge, attitudes, and roles, standardize programs, and inform policy development.

**Abstract:**

**Background/Objectives**: Developmental Language Disorder (DLD) affects approximately two children in every classroom and significantly impacts literacy development and academic achievement. Given the central role of language in learning, teachers are well-positioned to identify, support, and advocate for children with DLD through referrals, interventions, and inclusive curriculum delivery. However, evidence consistently indicates that teachers lack fundamental knowledge of DLD, highlighting an urgent need for targeted professional training. This study, conducted in Cyprus, aimed to (1) explore pre-school and primary school teachers’ knowledge and views regarding DLD and (2) synthesize an evidence-based checklist of essential topics for DLD teacher training. **Methods**: A total of 133 teachers completed an online questionnaire addressing three research questions: teachers’ knowledge of DLD and its characteristics; their attitudes toward DLD; and their perceptions of their role in supporting children with DLD. **Results**: Findings aligned with international trends, showing limited confidence in supporting students with DLD despite reasonable familiarity with the label and its core features. Teachers demonstrated a broad understanding of their supportive role but acknowledged knowledge limitations and requested structured professional development. Based on these findings and existing literature, the Basic-DLD Guide was created for researchers, practitioners, and continuing education providers, to inform the development of basic trainings. **Conclusions**: The study’s findings and the guide can have direct clinical significance, providing an evidence-informed foundation for designing structured professional training to improve identification and support for children with DLD in educational settings.

## 1. Introduction

Developmental Language Disorder (DLD) is a disorder that affects 1 in 14 children [1], causing difficulties in understanding and expressing language without an apparent and known reason. The language ability of affected children is found to be significantly lower than that of their peers. DLD impacts multiple, interconnected areas of language. For example, children with DLD often have difficulties with learning and recalling words (semantics), forming complex sentences and using grammatical structures correctly (syntax and morphology), and difficulties with speech sounds (phonology). DLD is formally diagnosed by speech and language therapists (SLTs) using diagnostic criteria that focus on persistent, significant language difficulties and their functional impact on everyday life. The onset of the disorder is not attributed to factors such as low intelligence, overt neurological condition, sensory organ damage, genetic syndrome, cognitive deficits, or environment [2]. Children with DLD face significant risks, such as poor academic outcomes (e.g., difficulties in reading, spelling, and mathematics) and mental health problems (e.g., anxiety, clinical depression) [3].

DLD is not a condition that can be prevented as it arises from complex neurodevelopmental factors that are not yet fully understood. However, its impact can be significantly reduced through early identification and evidence-based interventions tailored to the individual’s needs. With appropriate support, such as speech and language therapy, classroom accommodations, and caregiver and public education, individuals with DLD can improve their communication skills and reach their full potential. Unfortunately, this is not the case for many people with DLD who remain unidentified and do not have access to the required support services.

Based on the prevalence rate of 7.5% reported in the literature [1], it is expected that approximately 3500 children in Cyprus attending primary education and 2800 children attending general secondary education have DLD. Since the 1980s in Cyprus, SLTs have been providing their professional services to children with speech, language, and communication difficulties. These services are available in school settings, hospitals and private offices [4]. Nevertheless, identifying children with language difficulties in inter-linguistic settings, such as in Cyprus where the native language is the Cypriot Greek dialect and the official language of the school is Modern Greek [5], is complex, as there are no screening and assessment tools designed and standardized for this population [6].

The significant impact of the disorder on literacy skills and the importance of language skills in the learning process, underscores the positioning of DLD as a diagnosable clinical condition and an educational concern. It also highlights the multi-dimensional role of a teacher for the discussed population. While SLTs diagnose DLD and provide evidence-based intervention services for its remediation, teachers also play a critical role in supporting students with DLD, by providing direct or indirect support in the classroom, educating them in the curriculum, and collaborating with SLTs for improved learning outcomes. Additionally, teachers are often required to make referrals for the appropriate services regarding children at high risk for DLD. It is important to mention that in Cyprus, according to the Law of 1999 (113(I)/1999) on Special Education and Education of Children with Special Needs and its relevant amendments (69(I)/2001, 87(I)/2014, 166(I)/2020), teachers are expected to make referrals of children whose learning or other skills deviate from those expected for their chronological age to the Educational Psychology Service of the Ministry of Education, Sports and Youth or to the Provincial Committees for Special Education and Training, in order to evaluate, determine and receive the required services for them. If children with DLD are experiencing severe deviations, they may receive Special Education and/or Speech and Language Therapy services within the mainstream school, in the form of individual and/or group sessions [7].

To make appropriate and timely referrals, teachers must first understand what DLD is and how it manifests in the classroom. Over the past few years, we have seen an increase in studies examining teachers’ knowledge of DLD in various countries, to identify gaps and professional learning needs [8,9,10,11,12]. The current study extends such efforts to the Cypriot school context by (1) examining teachers’ knowledge and views about DLD and their role in supporting children with DLD, and (2) creating a universal guide for designing seminars and course introducing DLD to teachers.

### 1.1. Current Evidence on Teacher Knowledge of DLD

Teachers’ knowledge of DLD has been investigated in several countries. In England, teachers reported limited understanding of terminology used by SLTs and researchers (e.g., DLD) and they showed greater uncertainty about which language behaviours indicated speech versus language difficulties [9]. However, teachers were aware of the co-occurrence of language difficulties with academic difficulties, especially reading comprehension and writing. Teachers highlighted the lack of training and the need for tools for identifying children with language difficulties.

Findings from Ireland also highlight terminology-related challenges, as teachers referred to DLD using a variety of terms and expressed a lack of clarity regarding diagnostic terminology [12]. Teachers recognized their critical role in meeting the needs of children with DLD during school time and the importance of good interprofessional collaboration with SLTs and special educators.

In Australia, teachers demonstrated a mismatch between self-rated and demonstrated knowledge, with knowledge gaps primarily concerning the lifelong and persistent nature of DLD, even if direct or indirect intervention is provided, educational impacts of DLD, multilingualism, and cognitive referencing [10]. However, teachers recognized the value of DLD-friendly strategies in the classroom (e.g., pace, visual support systems) and trusting professional relationships with SLTs and special educators.

Ciullo and Hoover (2025) [8] adapted the survey conducted by Glasby et al. (2022) [10] to the US context. They found that while teachers demonstrated a good understanding of school activities that required increased language demands for children with DLD, many of them were not familiar with the term ‘developmental language disorder’ and reported similar misconceptions about the nature of DLD as their Australian counterparts, namely that children with DLD would “catch-up and match peers’ language skills—if provided with language intervention/therapy”. Like in other countries [10,12], many teachers in the US emphasized the importance of a team-based approach for meeting the multifaceted needs of children with DLD.

Finally, in Greece, half of the teachers did not provide a definition of DLD and many of them recognized their own knowledge limitations and need for further training [11]. Among the definitions provided, most focused on language deficits, which the authors deemed encouraging. Moreover, many teachers identified common language difficulties in DLD (e.g., vocabulary, syntax), and additional emotional, learning, and behavioural difficulties. Teachers also acknowledged the importance of working with other specialists to efficiently support children with DLD.

### 1.2. The Importance of Improving Teacher Knowledge of DLD

Public schools are a primary setting for the early identification and support of children with DLD [13,14], and teachers are required to refer these children to appropriate services, as defined in Cyprus’ special education laws. Moreover, teachers spend many hours every day with children with DLD and they must have the knowledge and skills to understand how language difficulties manifest in the classroom and how instruction can be tailored to children’s needs. How will teachers ‘see’ DLD, if they do not know about it? [15]. As with any type of change, improving teachers’ knowledge of DLD is usually the first line of defence when attempting to improve school-based practices.

Training on DLD has been shown to increase teacher knowledge. In a recent study conducted by Foley et al. (2023) [16], teachers reported significant changes in their awareness of language development and disorders, after participating in a whole-school program that included six workshops. In addition, many teachers used the knowledge and skills acquired during the workshops to create DLD-friendly classroom environments. It is worth highlighting that the program implemented by Foley et al. also included follow-up support for teachers (e.g., classroom observation, support from SLTs, sharing of resources) and training to conduct language screening. This points to the importance of systematic, continuous, and collaborative efforts for improving educational practices for children with DLD [13,17].

### 1.3. Current Study

Evidence from other countries suggests that teachers often struggle with basic knowledge about DLD (e.g., terminology, symptomatology, educational impacts, DLD across the lifespan), emphasizing the need for targeted training opportunities [8,9,10,11,12].

In this first study conducted in Cyprus, we aimed to extend such efforts in two ways. First, we explored pre-school and primary school teachers’ knowledge and views about DLD, through the following research questions:

*1.*  *What is teachers’ knowledge of DLD and its characteristics?*
*2.*  *What are teachers’ attitudes about DLD?*
*3.*  *What are teachers’ views of their role in supporting children with DLD?*

Second, we synthesized our findings and those of others into a practical, evidence-informed checklist that outlines essential topics for DLD training. Continuing education providers can use such a checklist to develop basic seminars and workshops, helping teachers understand DLD in the context of school-based practice.

## 2. Materials and Methods

### 2.1. Participants

A total of 133 teachers, 14 pre-school and 119 mainstream primary school teachers, working in public education, took part in the current research. Participation was voluntary, and the questionnaire was shared online, which could have led to self-selection bias: teachers who are more aware of DLD might have been more inclined to participate, possibly overestimating the familiarity. The participating educators originated throughout the country of the Republic of Cyprus, specifically 16 from Paphos, 93 from Limassol, 4 from Larnaca, 15 from Nicosia, and 5 from Famagusta. In terms of gender, 120 were female and 13 were male. Additional demographic information, including education levels, grades taught, and years of teaching experience is provided in Table 1. It is noted that 70% of the participants (n = 93) were from Limassol, reflecting geographic concentration of recruitment around the first author’s institution. Findings may more closely reflect the professional profile of Limassol-based teachers.

### 2.2. Materials

The investigation of teachers’ knowledge and views on DLD was conducted using an open online questionnaire on the Google Forms platform. A significant factor in choosing this platform was its integrated functions that facilitate recording results and data analysis.

The online questionnaire for the present survey consisted of 31 questions (including sub-questions), mostly closed-ended, and was divided into five parts. Before completing the questionnaire, the participants received a briefing about the research study (topic and purpose) and were given the terms used in the literature as synonyms or similar for DLD, such as Specific Language Disorder, Specific Language Impairment, Primary Language Disorder, and language disorder of comprehension and expression. The questionnaire was organized into five parts: demographic background, teachers’ knowledge of DLD and its characteristics, their attitudes toward DLD, their perspectives on their role in supporting children with DLD, and their professional needs. Since the questionnaire covered a broader range of topics than the specific aims of this paper, only the relevant sub-findings are reported here, corresponding directly to the three research questions outlined earlier. This paper focuses exclusively on the findings relevant to the three research questions.

The questionnaire is an adaptation of the instrument utilized by Thordardottir, Topbaş, and the Working Group 3 COST Action IS1406 [18] to assess the public’s understanding of the characteristics and causes of Developmental Language Disorder (DLD). The modifications implemented were varied, and the target populations of the two questionnaires differed with respect to the study’s objectives. Items concerning general awareness of DLD among the public were preserved with minor linguistic adjustments, whereas items specific to teachers’ professional roles, classroom practices, and professional development were newly developed. Additionally, items pertaining to Cypriot legislative requirements were incorporated.

Prior to administration, content validity was assessed by 4 SLTs who evaluated item clarity, relevance and professional appropriateness. Their feedback informed minor revisions to item wording and response options. The original instrument has demonstrated utility across diverse populations and contexts, having been used in a large-scale European survey on public awareness of childhood language impairment [18] a multi-country study on public awareness of developmental language disorder (DD) in Croatia, Italy, and Slovenia [19], and a study examining foreign language teachers’ awareness of language difficulties [20]. These applications support the instrument’s credibility and adaptability for both public and professional groups. However, as the adapted version was not subjected to formal psychometric validation within the Cypriot context, the findings should be considered exploratory. Specifically, the lack of established reliability and construct validity coefficients for the adapted instrument limits the certainty with which it measures constructs such as teachers’ knowledge and attitudes toward DLD. Therefore, caution is warranted when generalizing these findings beyond the current sample or to teacher populations in other educational and cultural contexts. Future research should conduct comprehensive psychometric validation of adapted instruments prior to broader application to enhance the comparability and transferability of findings across settings.

### 2.3. Recruitment and Consent Procedures

Potential participants were contacted via email, which included a link to the online questionnaire and an invitation to take part in the survey, accompanied by a detailed description of the study and its purpose. Additionally, the questionnaire and survey information were disseminated through social media and the website of the first author’s research laboratory (https://www.cut.ac.cy/faculties/hsc/reh/research/research-labs/epilog/) (accessed on 28 April 2026), based at Cyprus University of Technology.

The study was approved by the National Bioethics Committee of Cyprus (ΕΕΒΚ ΕΠ 2018.01.82). A prerequisite for teachers’ participation in the survey was their consent. Therefore, before beginning the first part of the online questionnaire, participants were provided with information about the survey, including the option to give consent or withdraw at any time, as participation was voluntary. If a teacher chose not to participate, they could click the “NO” option to exit the online window.

### 2.4. Data Analysis

Closed-ended responses were analysed using descriptive statistics. Open-ended responses were analysed using qualitative content analysis [21] employing an inductive approach to allow thematic categories to emerge from the data. The thematic analysis was conducted collaboratively by the first and second authors, both with expertise in speech and language therapy. Responses were read in full before coding began, and thematic categories were developed through ongoing discussion and consensus between the two coders. The final categorisation was reviewed by the third author, who examined the coherence and mutual exclusivity of the categories and confirmed their appropriateness. While formal inter-rater reliability statistics were not calculated, the consensus coding approach and independent third-author review served as procedural safeguards for the credibility and consistency of the analysis.

## 3. Results

In the first section, we report descriptive statistics to answer our primary research questions: (1) Teachers’ knowledge of DLD and its characteristics; (2) Teachers’ views about DLD; and (3) Teachers’ perceptions of their role in supporting children with DLD. In the second section, we present Basic-DLD, a simple guide to develop basic seminars on DLD for teachers (see Appendix A).

### 3.1. Teachers’ Knowledge of DLD

#### 3.1.1. Familiarity with Language Milestones

**Figure 1:**
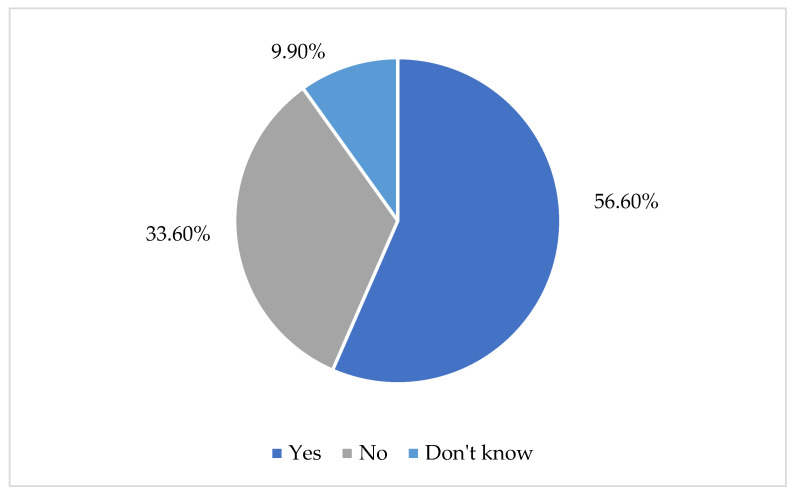
Teachers’ knowledge of language milestones.

Teachers were asked whether they are familiar with language development milestones. More than half (56.5%) reported that they are familiar with these milestones, while 33.6% stated that they are not. Additionally, 9.9% indicated that they were unsure, reflecting some uncertainty about whether they are adequately informed or not (see Figure 1).

#### 3.1.2. Familiarity with the Term ‘DLD’

**Figure 2:**
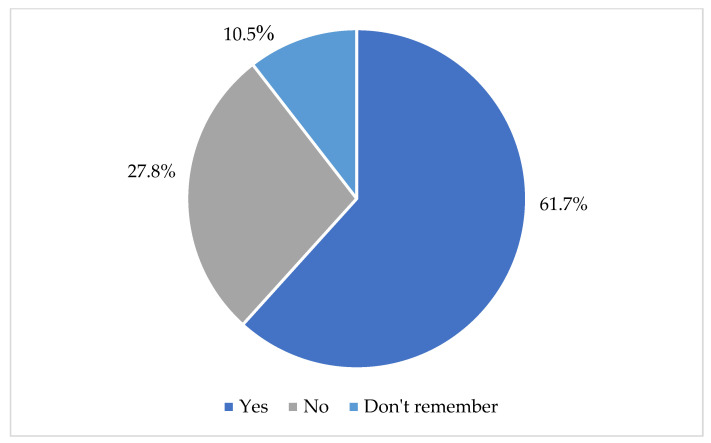
“Have you heard of the term DLD (or its synonyms) before?”.

In response to the question, “Have you heard of the term DLD (or its synonyms) before?” 27.8% of participants indicated that they were unfamiliar with the term, and 10.5% reported that they could not recall whether they had encountered it (see Figure 2).

**Figure 3:**
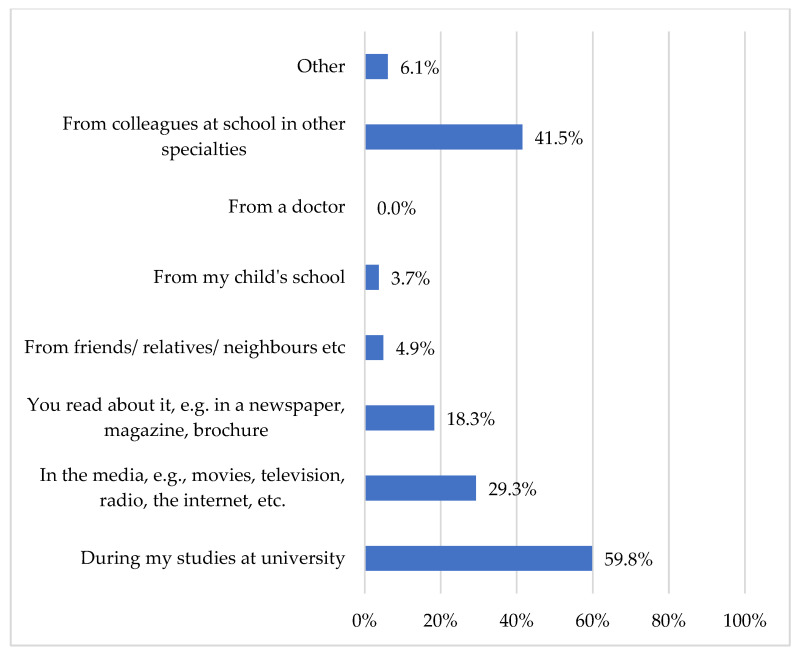
“Where have you heard of the term ‘DLD’?”.

Participants who reported prior exposure to the term DLD were asked to identify the source of their initial encounter. The majority reported their first exposure during university studies, whereas 41.5% reported learning about the term from other professionals at school. A smaller proportion cited mass media and print media as their initial sources (see Figure 3).

#### 3.1.3. Definition/Description of DLD

Participants were then asked to provide a definition/description of DLD. Seventy-seven participants answered this question, and the analysis of their responses revealed eight main themes. It should be noted that several responses fall into more than one thematic category, as they include multiple dimensions of the disorder.

***Theme 1: Deviation from the expected age level***. The most frequently mentioned dimension (n = 27) concerns the language disorder as a delay or deviation compared to peers. Participants described the disorder as “impaired development in spoken and/or written language relative to the average for the child’s age” and pointed out that the child “has not acquired language skills to a satisfactory degree for their age.”

***Theme 2: Difficulties in language and speech production.*** Many participants (n = 35) focused on the expressive dimension of the disorder, i.e., the inability to produce spoken or written language. Typical references include “difficulty in speech production” and the observation that the child “cannot express themselves in words or speak properly.”

***Theme 3: Difficulties in understanding speech.*** Several responses (n = 22) highlighted the difficulties in language comprehension. Participants reported “difficulties in verbal expression and/or understanding speech” and noted that the child “has difficulty understanding the meaning of words” or “does not understand what they read.”

***Theme 4: Specific language skills affected.*** Some participants (n = 18) identified specific language areas affected. In the area of articulation and phonology (n = 8), “articulation problems” and “phonological weaknesses” were mentioned. In terms of vocabulary (n = 7), “poor vocabulary” and “difficulty acquiring vocabulary” were highlighted. In the area of syntax (n = 6), “difficulties in constructing sentences” and “syntactic errors” were reported, while in written language (n = 5), problems in “decoding and understanding written language” were highlighted.

***Theme 5: Exclusion of other causes.*** An important finding concerns the reference by some participants (n = 5) to the diagnostic criterion of exclusion of other conditions. Specifically, they reported that the disorder “is not due to mental retardation or any other neurological cause” and that it is “a disorder that is not due to or associated with other conditions.”

***Theme 6: Impact on the child’s life.*** Few participants (n = 8) recognized the disorder’s impact on areas beyond language. They reported that the disorder “impedes smooth communication, education, and overall development” and that “learning, literacy, and social behaviour are affected.” In addition, it was pointed out that the disorder creates “problems in the academic, social, and psychological spheres.”

***Theme 7: Causal factors.*** Only a few participants (n = 4) mentioned possible causes of the disorder. These included “environmental or biological factors,” “speech pathology or other reasons,” and “lack of stimulation.”

***Theme 8: Confusion with other disorders***. Finally, some responses (n = 5) showed confusion between language disorder and specific disorders or symptoms. Typical examples include identification with “dyslexia,” “selective mutism,” and “stuttering.”

#### 3.1.4. Symptoms of DLD

Participants were subsequently asked to select symptoms they believed described DLD based on their existing knowledge. Teachers most frequently identified delayed language milestones (56.4%), difficulty expressing ideas (62.4%), problems with syntax (60.2%), trouble telling stories or events (56.4%), incorrect grammar (55.6%), and trouble with sound production (12.0%) as primary features. Additionally, several effects typically associated with developmental language disorder (DLD), including reading difficulties (56.4%), writing challenges (54.9%), and low self-esteem (51.9%), were also attributed to DLD by participants (see Table 2).

#### 3.1.5. Specialists Qualified to Recognize DLD

**Figure 4:**
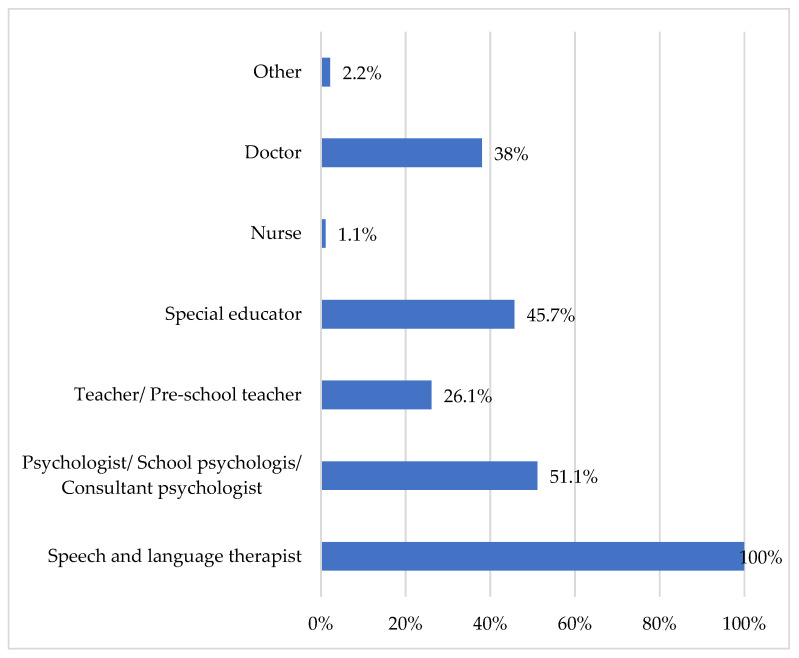
“Which specialists are qualified to recognize DLD in children?”.

To assess teachers’ knowledge and attitudes regarding the management and intervention of children with DLD, participants were asked to identify which specialists are qualified to recognize DLD in children. All respondents indicated that SLTs possess the necessary expertise to determine whether a child has a speech or language disorder. Additional specialists identified as capable of recognizing these disorders included special education teachers (45.7%), psychologists (51.1%), teachers (26.1%), and doctors (38%) (see Figure 4).

#### 3.1.6. Service Access for Children with DLD in Cyprus

**Figure 5:**
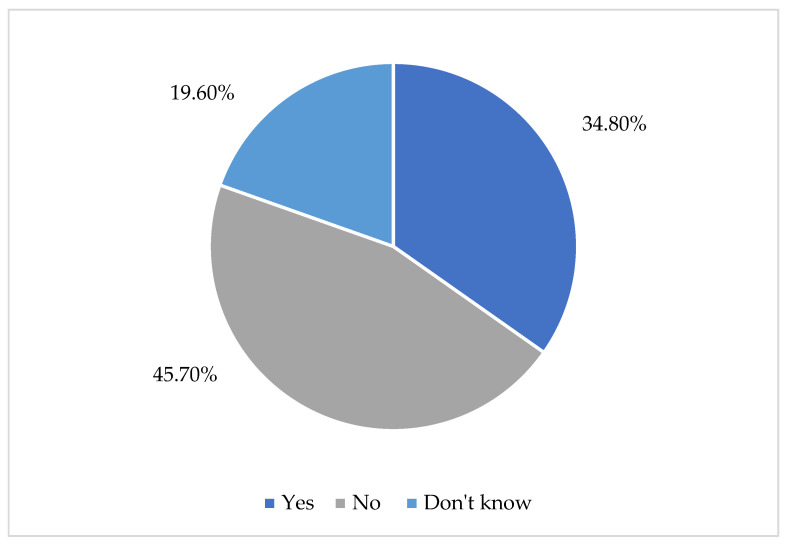
“Can all children with DLD in Cyprus access intervention services?”.

Participants were asked whether all children with DLD in the country can have access to intervention services. Many of the teachers indicated that not all children have such access (45.7%). In contrast, 34.8% of respondents reported that services are available to all children, while 19.6% selected “I don’t know” (see Figure 5).

### 3.2. Teachers’ Attitudes About DLD

#### 3.2.1. General Attitudes About DLD

Participants were presented with a sentence completion task (“If a child has DLD…”) with preformulated response options, designed to elicit their attitudes toward children with DLD. Analysis of responses reveals predominantly positive attitudes among the sample. More than half of the participants (66.2%) endorsed the importance of environmental understanding and accommodation for children with DLD, while an even greater proportion (84.2%) expressed support for modifying established routines to promote the child’s participation in activities (see Table 3).

#### 3.2.2. Attitudes About Prevention of DLD

**Figure 6:**
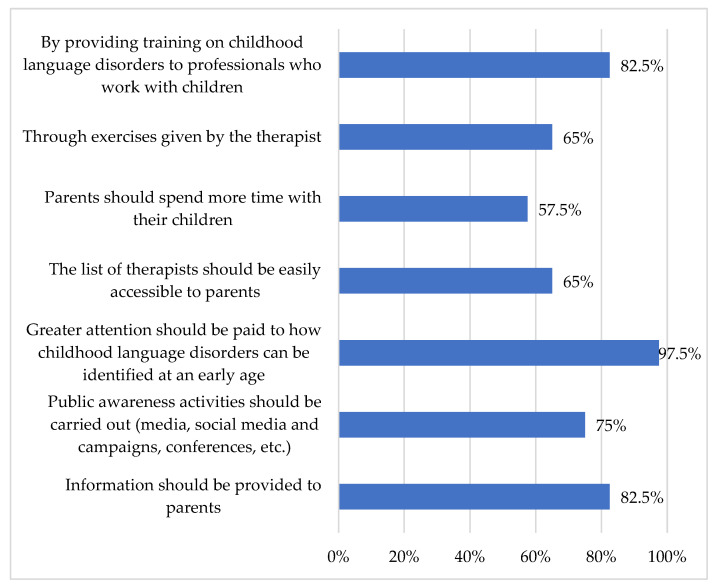
“How can DLD be prevented?”.

In response to questions regarding the prevention of DLD (How can DLD be prevented?), most teachers (97.5%) emphasized the significance of early identification of the disorder. Additionally, many (82.5%) underscored the importance of providing training for professionals who work with children with DLD and parents (see Figure 6).

### 3.3. Teachers’ Views About Their Role in Supporting Children with DLD

#### 3.3.1. Knowledge to Teach Children with DLD

**Figure 7:**
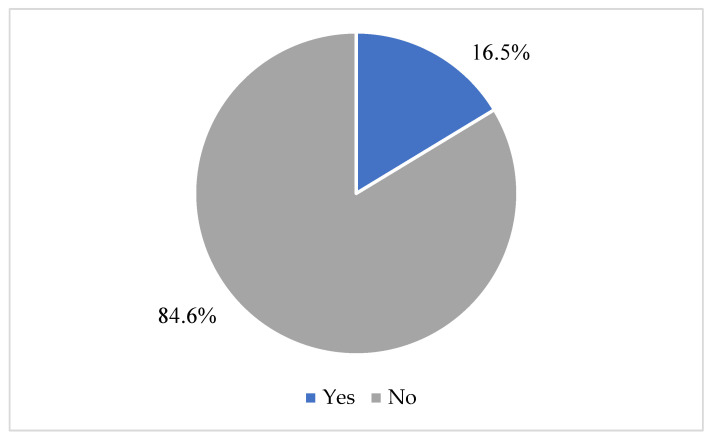
“Do you consider that you have the necessary knowledge to teach children with DLD in the classroom?”.

In response to the question, “Do you consider that you have the necessary knowledge to teach children with DLD in the classroom?”, most teachers (84.6%) indicated that they do not possess sufficient knowledge to teach children with DLD (see Figure 7).

#### 3.3.2. Effective Methods to Improve Knowledge of DLD

**Figure 8:**
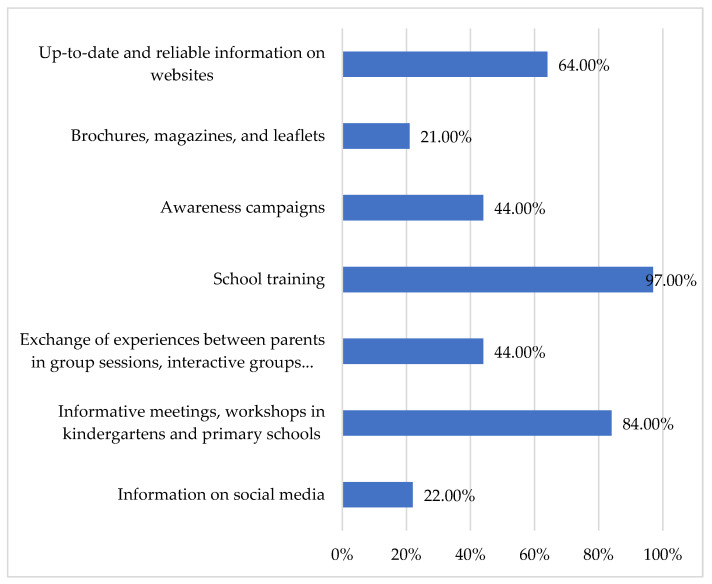
Effective methods to improve knowledge of DLD.

Participants were asked to identify effective methods for training about DLD. Participants’ responses reveal a clear preference for formal, school-supported professional development (see Figure 8). The overwhelming majority (94%) indicated that continuing education provided within the school context would be beneficial. Furthermore, 85.2% endorsed informative sessions as a useful method, and 67.1% expressed the need for access to current research and information. Notably, fewer than half of the participants perceived peer-to-peer information exchange or broader awareness campaigns as effective means of acquiring knowledge about DLD.

#### 3.3.3. General Roles in Supporting Children with DLD

**Figure 9:**
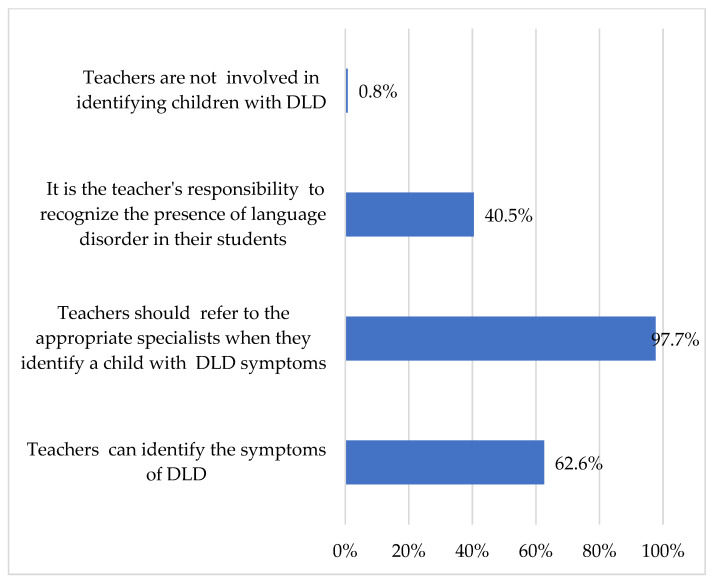
Teachers’ roles in supporting children with DLD.

Participants demonstrated an understanding of their responsibilities as teachers and acknowledged their capacity to refer children to relevant specialists (97.7%). 62.6% of participants expressed confidence in identifying children who may be experiencing DLD (see Figure 9).

#### 3.3.4. Roles in Teaching Children with DLD

**Figure 10:**
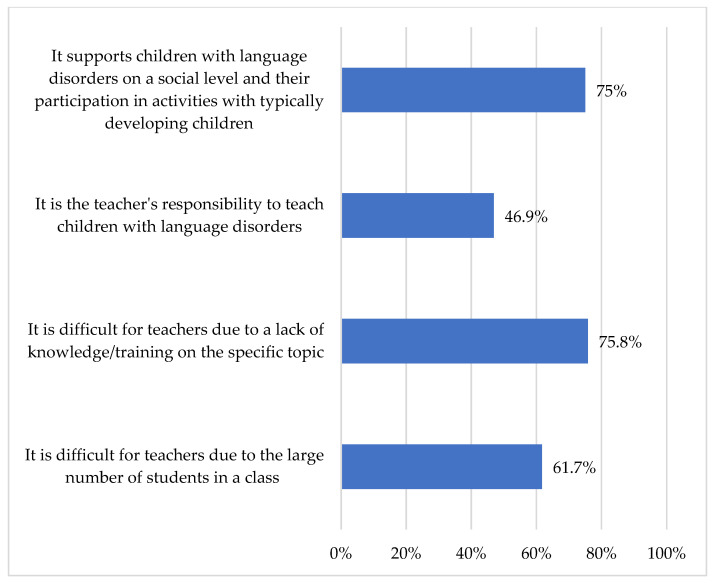
Teachers’ roles in teaching children with DLD.

Less than half of participants agreed with the statement, “It is the teacher’s responsibility to teach children with language disorders.” Notably, 75.8% attributed this reluctance to a perceived lack of necessary knowledge. Many teachers also reported difficulties in supporting these children due to large class sizes. Nevertheless, 75% of participants indicated that their role includes facilitating the participation of children with DLD in peer activities (see Figure 10).

#### 3.3.5. Roles in Intervention and Interprofessional Collaboration

**Figure 11:**
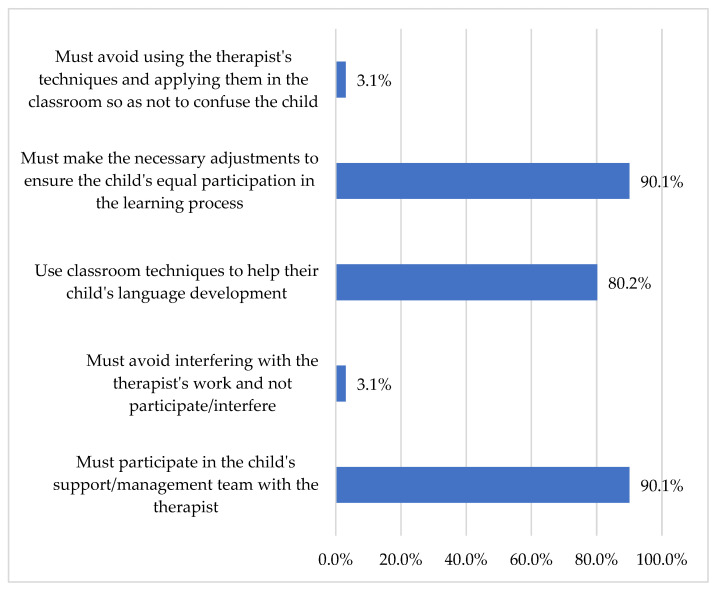
Teachers’ roles in intervention and interprofessional collaboration.

Then, teachers were asked, “What do you think is the role of teachers in the process of treatment or intervention of their students who receive services from an SLT?” The majority of teachers (90.1%) indicated that their role in supporting a child with DLD who receives speech therapy involves participation in an interdisciplinary team dedicated to assisting children with language difficulties. They (90.1%) further indicated that it is their responsibility to implement modifications that promote equal participation for all students and to employ effective instructional strategies to support the language development of children with DLD (Figure 11).

### 3.4. Basic DLD: A Guide to Develop Basic DLD Training

Based on our findings and those of others [8,9,10,11,12], we developed a guide to help researchers, practitioners, and continuing education providers create basic DLD training for teachers (see Appendix A).

The guide is divided into three primary domains. The first domain is ‘Knowledge of DLD’ and includes the following subdomains: (a) language developmental and typical milestones in school-age children, (b) DLD terminology, (c) causes of DLD, (d) DLD characteristics and symptoms, (e) impact of DLD on children’s lives, (f) assessment and diagnosis of DLD, and (g) knowledge of relevant policies and laws on the rights of children with language difficulties.

The second domain is ‘Attitudes and Views about DLD’ and includes the following subdomains: (a) stereotypes around DLD, (b) participation and inclusion for children with DLD, and (c) tailoring classroom environments to better support children with DLD.

The third domain is ‘Roles in supporting children with DLD and includes the following subdomains: (a) teachers’ roles in early identification of DLD and prevention, (b) teachers’ roles in supporting children with DLD in the classroom, and (c) teachers’ roles in interprofessional collaborations.

For each domain, we have provided a list of useful and, mostly, open access resources. This list is not exhaustive as there are many more high-quality resources available online. However, it provides a starting point towards the creation and delivery of evidence-based and practical information.

## 4. Discussion

This study investigated pre-primary and primary school teachers’ knowledge, attitudes, and views regarding DLD, striving to identify relevant training topics for professionals supporting students with DLD and to improve educational provision for these children in mainstream settings. A primarily descriptive approach was adopted, which is appropriate given the exploratory nature of the study and the limited prior research available within the Cypriot educational context. Rather than testing causal or predictive relationships, the study sought to establish a foundational understanding of where teachers currently stand in terms of awareness, attitudes, and perceived professional needs, a necessary first step before more analytically rigorous investigations can be meaningfully pursued. The outcomes contribute to the expanding international literature on teachers’ understanding of DLD, common misconceptions, and the role of training in improving school-based support [8,9,10,11,12,16].

The Cypriot findings are notable when compared directly with the international literature. Unlike teachers in Greece [11], where half did not provide any definition of DLD, the majority of Cypriot participants produced substantive descriptions, suggesting comparatively greater baseline awareness, possibly reflecting the impact of a recent national awareness campaign and the growing visibility of SLT services within Cypriot schools. Conversely, Cypriot teachers’ uncertainty about exclusionary criteria, the lifelong nature of DLD, and deeper conceptual understanding remains a shared challenge regardless of the awareness level. This distinction between surface familiarity and functional knowledge is important. Teachers may recognise a label without understanding its diagnostic boundaries or classroom characteristics, a gap that basic training must specifically address.

Teachers are typically responsible for initiating referrals within public education systems, making it necessary to understand how they conceptualize DLD. If teachers are unable to recognize language difficulties as they manifest in classroom activities, early identification is unlikely [15]. The Cypriot legislative framework explicitly designates teachers as gatekeepers to specialist services, rendering knowledge of DLD necessary for equitable access to support.

The pattern identified in this study closely reflects international findings. Teachers most frequently describe DLD as deviations from age expectations, emphasizing expressive language difficulties, grammar, and sentence formulation. Many also recognized comprehension problems and connections with literacy, particularly in reading and writing. Comparable trends have been documented in other countries (e.g., Australia: [10]; US: [8]; Greece; [11]), in which teachers tend to identify apparent behaviors and academic consequences but remain uncertain about terminology, etiology, and diagnostic boundaries.

Deeper aspects of the disorder were seldom addressed. Few participants mentioned exclusionary criteria, the developmental and continuing nature of DLD, or its possible long-term psychosocial effects. Confusion with other conditions, such as dyslexia or stuttering, was also apparent. These findings are consistent with Glasby et al. (2022) [10] and Ciullo and Hoover (2025) [8], who reported misconceptions about whether children “catch up” after intervention and a limited understanding of the lifespan implications of DLD.

Although teachers recognized many classroom manifestations of language difficulty, most did not feel confident in their ability to teach these students effectively. The majority reported insufficient knowledge. This finding resonates with international calls for clearer guidance, shared terminology, and accessible tools that translate research into practical decision-making models [8,9].

Despite these gaps, the current results may indicate gradual improvement compared with older literature. More than half of the participants reported familiarity with the label DLD and could identify several core linguistic features. Considering recent awareness initiatives and policy attention in Cyprus [7], this suggests that national efforts to standardize terminology and increase visibility may already be influencing professional understanding. Comparable terminology-related uncertainty has been reported in Ireland [12], indicating that such changes require time and sustained reinforcement.

The most encouraging finding relates to teachers’ attitudes and views about their roles in supporting children with DLD. Participants supported inclusive practices, emphasized acceptance and environmental adjustments, and rejected stigmatizing beliefs. They identified themselves as partners in multidisciplinary collaboration and endorsed the value of working closely with SLTs and other specialists. Similar positive orientations toward teamwork have been reported internationally [8,10,12]. However, willingness was accompanied by insecurity. Teachers expressed greater confidence in referring students than in recognizing the signs of DLD or enacting targeted pedagogical strategies. This inconsistency between motivation and preparedness is a well-established theme in DLD research [22,23]. Educators require clearer knowledge, systematic support, and practical classroom tools.

Participants’ strong preference for formal, school-based professional development is particularly informative. Teachers are not simply aware of their limitations. They are explicitly requesting structured, authoritative, and ongoing opportunities to improve. Evidence from intervention studies supports this direction. For example, Foley et al. (2023) [16] demonstrated that sustained, whole-school programs accompanied by follow-up assistance and collaboration with SLTs can produce measurable improvements in knowledge and classroom practice. Short-term awareness-raising is unlikely to be sufficient; systematic and continuous partnerships are required [13,24].

### 4.1. Recommendations for Using the Basic-DLD Guide

As schools represent the primary environment for early identification (considering that most children with DLD attend public schools) [14], strengthening teacher knowledge is a critical first step for enhancing outcomes for children with DLD. Responsibility should be distributed across the professional continuum. Initial teacher education should provide a solid conceptual foundation in language development and disorders, while in-service systems should offer opportunities to revisit and deepen this knowledge as research evolves [10,25].

With the increase in DLD awareness campaigns worldwide, we notice an increase in demand for basic and advanced training for teachers. The Basic-DLD Guide includes suggested topics that aim to provide a solid foundation in language development and DLD, such as causes and characteristics of DLD, implications for diagnosis, common misconceptions around DLD, and teachers’ essential role in early identification and support of children with DLD (see Appendix A). These topics are critical for helping teachers in Cyprus and worldwide “see” the experiences of approximately two children with DLD in their classroom [1], understand their unique role in creating effective pathways for support, and inspire meaningful changes in practice that benefits all learners.

Researchers, practitioners, and training providers can use the Basic-DLD guide as a starting point for creating and tailoring learning content to their educational contexts. Contextual adaptation of the guide is essential. A substantial proportion of DLD research originates in English-speaking countries and its direct application to other linguistic and educational contexts may be limited. Training providers must critically evaluate which research findings are transferable and which require re-interpretation considering local linguistic characteristics. For example, an important topic under ‘Domain 1: Knowledge of DLD’ is ‘DLD characteristics and symptoms’. While there are universal DLD characteristics (e.g., poor sentence repetition), some aspects of DLD may differ across languages (e.g., grammar or morphosyntax). Additionally, the availability and validity of assessment tools vary significantly across languages and dialects [6]. Contextual adaptation of the guide is also important when addressing topics such as early identification, intervention, classroom support, and interprofessional collaboration. For example, in some countries, classroom teachers may play a formal role in referrals under current legislation, whereas in others they might not. Effective foundational training in DLD requires alignment with a country’s linguistic and cultural profiles, national policies, and teachers’ professional responsibilities within educational systems.

### 4.2. Limitations

Some methodological constraints should be acknowledged. First, data were collected via self-report, which may not accurately reflect actual teacher knowledge or classroom practices. Second, voluntary online participation can introduce self-selection bias, with teachers more interested in DLD more likely to take part. Third, social desirability effects cannot be ruled out, particularly for attitudinal items relating to inclusive practices. Fourth, the adapted questionnaire has not been formally validated psychometrically within the Cypriot context, which limits confidence in its construct validity. Lastly, because most participants are from Limassol, the findings may be less representative and less applicable to other regions in Cyprus.

### 4.3. Future Directions

The primarily descriptive approach adopted in this study was appropriate given its exploratory objectives and the limited prior research in the Cypriot context. Nevertheless, the findings highlight the need for future research that advances beyond foundational description toward more analytically rigorous examination of the relationships among teachers’ knowledge, attitudes, and professional needs regarding DLD. As the current results do not establish directionality or causal links between teacher knowledge and classroom practice, subsequent studies should employ more robust research designs, such as inferential and longitudinal methods, to investigate how these variables interact and influence each other over time. Future research should also incorporate stratified sampling strategies and utilize formally validated instruments within the Cypriot context, ensuring comprehensive psychometric validation of any adapted tools prior to broader implementation. These methodological enhancements will improve the comparability and transferability of findings across different settings. Building on these improvements, further research should empirically validate the Basic-DLD guide and assess its practical impact. Specifically, studies are required to determine whether the guide effectively addresses teachers’ learning needs or necessitates further refinement. Additionally, research should evaluate whether providing teachers with structured, evidence-based knowledge about DLD, as outlined in the Basic-DLD guide, leads to measurable improvements in identification and support practices. This includes assessing whether increased teacher knowledge results in more accurate and timely referrals for assessment and encourages the adoption of DLD-supportive strategies in the classroom.

## 5. Conclusions

The study demonstrates that although teachers are generally familiar with the term DLD and its fundamental characteristics and consequences, they lack adequate knowledge and confidence to accurately identify the disorder and support affected children. These findings align with international literature and highlight the need for structured, evidence-based professional training tailored to teachers’ specific requirements.

A key contribution of the study is its reliance on empirical data. Instead of developing educational content based on theoretical assumptions, the findings identify gaps in knowledge, uncertainties in attitudes, and the role of teachers in supporting DLD. The Basic-DLD Guide embodies this approach by organizing content into three documented domains: knowledge about DLD, attitudes and perceptions, and teachers’ roles in supporting children. Effective capacity building necessitates targeted training and professional development programs.

The implications of this study extend beyond the classroom. Teachers equipped with appropriate knowledge and practical strategies can substantially contribute to the early detection and support of children with DLD, thereby enhancing their literacy, academic performance, and social integration. Investment in teacher training represents an investment in early and effective intervention. Attention should also be directed to the role of policymakers. The study data provide a clear foundation for integrating specialized content on DLD into initial and ongoing teacher education programs. Training providers, whether in higher education or professional bodies, can utilize tools such as the Basic-DLD Guide to establish training standards, evaluate existing programs, and promote interdisciplinary collaboration. When policies do not explicitly reference DLD, affected children remain overlooked within systems designed to support them.

In conclusion, enhancing recognition and support for children with DLD requires systematic needs assessment, evidence-based training development, and responsive policies. The findings and the Basic-DLD Guide aim to serve as valuable resources for researchers, professionals, educators, and policymakers, ensuring that every child with DLD receives timely, evidence-based, and inclusive support.

## Figures and Tables

**Table 1:** Demographic information of participants.

Category	Subcategory	Number of Participants
School Level	Pre-school	14
	Primary	119
District (Residence)	Paphos	16
	Limassol	93
	Larnaca	4
	Nicosia	15
	Famagusta	5
Age Group	20–35 years	17
	35–50 years	96
	50–65 years	20
Gender	Female	120
	Male	13
Highest Education	Bachelor’s degree	47
	Master’s degree	82
	Doctoral degree	4
Country of Studies	Cyprus	90
	Greece	10
	UK	1
	France	1
	Bulgaria	1
Primary Grades Taught (*only among primary teachers*)	A or B	34
	C or D	24
	E or F	39
	Mixed/Multiple grades	22
Years of Teaching Experience	>20 years	60
	≤20 years	73

**Table 2:** Teachers’ knowledge of DLD symptoms.

Symptoms	Yes	No	Don’t Know
Delay in saying his first words	56.4%	2.3%	5.3%
Difficulty saying words and pronouncing sounds	61.7%	1.5%	3.8%
Difficulty putting his ideas into sentences	62.4%	0.8%	4.5%
Difficulty choosing the correct grammatical forms of words (e.g., correct word endings)	55.6%	-	10.5%
Difficulty forming correct syntactic sentences	60.2%	0.8%	6.0%
Difficulty understanding instructions	51.1%	2.3%	10.5%
Difficulty telling stories	56.4%	2.3%	6.0%
Difficulty reading	56.4%	3.0%	4.5%
Difficulty writing	54.9%	2.3%	9.8%
Difficulty learning more than one language	29.3%	4.5%	29.3%
Difficulty creating or understanding jokes	20.3%	9.0%	31.6%
Difficulty acquiring knowledge at school (kindergarten, elementary school, middle school)	32.3%	18.0%	12.8%
Difficulty with mathematics	21.1%	22.6%	18.0%
Difficulty making friends	26.3%	27.1%	7.5%
Difficulty with social skills	35.3%	17.3%	8.3%
Teased and bullied by peers	30.1%	20.3%	11.3%
Low self-esteem	51.9%	4.5%	7.5%
Rarely invited to parties or social activities by friends	16.5%	25.6%	18.8%
Difficulty finding work as an adult	22.6%	12.8%	27.1%

**Table 3:** “If a child has DLD”.

If a Child Has DLD	Yes	No	Don’t Know
…you need to talk to someone who deals with or treats Developmental Language Disorder (other than a doctor or specialist).	82.70%	11.30%	8.30%
…this makes you, the child, and his/her family feel ashamed.	1.50%	88%	7.50%
…people in the child’s environment (classmates/friends/family) must understand and accept the child.	84.20%	9%	5.30%
…people will offer help.	66.90%	13.50%	17.30%
…the child must stay at home to hide his or her problem.	-	96.20%	1.50%
…strangers react inappropriately when the child speaks.	26.30%	37.60%	33.80%
…people in the child’s environment must change their habits to ensure that the child participates in various activities.	66.20%	12.80%	18.80%

## Data Availability

The raw data supporting the conclusions of this article will be made available by the authors upon reasonable request.

## Abbreviations

The following abbreviations are used in this manuscript:

DLD	Developmental Language Disorder
SLT	Speech–Language Therapist

## Appendix A

**Table A1:** Basic-DLD Guide: A guide to develop basic DLD training for teachers.

Learning Topics	Useful Resources ^1^
**Domain 1: Knowledge of DLD**	
1a. Language development and typical milestones in school-age children	Look for language-specific resources (e.g., Greek, English, French, Spanish, Arabic) https://academy.pubs.asha.org/2025/03/special-issue-highlights-arabic-language-acquisition/(accessed on 26 April 2026)
1b. DLD terminology	Phase 2 of CATALISE: a multinational and multidisciplinary Delphi consensus study of problems with language development: Terminology https://acamh.onlinelibrary.wiley.com/doi/full/10.1111/jcpp.12721 (accessed on 26 April 2026)
	Article about DLD terminology in Greek https://speechtherapy.org.cy/wp-content/uploads/2023/09/%CE%95%CE%BD%CE%B7%CE%BC%CE%B5%CF%81%CF%89%CF%84%CE%B9%CE%BA%CE%BF%CC%81-%CE%B1%CC%81%CF%81%CE%B8%CF%81%CE%BF-%CE%91%CE%93%CE%94_%CE%A4%CE%B5%CE%BB%CE%B9%CE%BA%CF%8C.pdf (accessed on 26 April 2026)
	The many terms used for DLD (in the English language) https://www.dldandme.org/all-articles/the-many-terms-used-for-dld (accessed on 26 April 2026)
	Discussion of terminology in other languages https://radld.org/about/dld/dld-fact-sheet/(accessed on 26 April 2026)
1c. Causes of DLD	Phase 2 of CATALISE: a multinational and multidisciplinary Delphi consensus study of problems with language development: Terminology https://acamh.onlinelibrary.wiley.com/doi/full/10.1111/jcpp.12721 (accessed on 26 April 2026)
1d. DLD characteristics and symptoms	Developmental Language Disorder https://www.nidcd.nih.gov/health/developmental-language-disorder (accessed on 26 April 2026)
	Identifying and describing developmental language disorder in children https://onlinelibrary.wiley.com/doi/pdfdirect/10.1111/1460-6984.12984 (accessed on 26 April 2026)
	Developmental Language Disorder and autism: Commonalities and differences on language https://www.mdpi.com/2076-3425/11/5/589 (accessed on 26 April 2026)
	DLD and dyslexia https://thedldproject.com/dld-dyslexia/(accessed on 30 April 2026)
	DLD and ADHD https://thedldproject.com/dld-adhd/(accessed on 26 April 2026)
1e. Impact of DLD on children’s lives (i.e., educational, social, emotional, mental, economic impact)	How we fail children with Developmental Language Disorder https://pubs.asha.org/doi/10.1044/2020_LSHSS-20-00003 (accessed on 26 April 2026)
	A systematic review of the academic achievement of primary and secondary school-aged students with developmental language disorder https://journals.sagepub.com/doi/full/10.1177/23969415221099397 (accessed on 26 April 2026)
	A first-person account of Developmental Language Disorder https://pubs.asha.org/doi/10.1044/2023_AJSLP-22-00247 (accessed on 26 April 2026)
	Education and employment outcomes of young adults with a history of developmental language disorder https://onlinelibrary.wiley.com/doi/10.1111/1460-6984.12338 (accessed on 26 April 2026)
1f. Assessment and diagnosis of DLD	The DLD Diagnostic Toolbox https://www.uwo.ca/fhs/lwm/news/2020/06_24_BlogIntroduction.html (accessed on 26 April 2026)
	Diagnosing and treating Developmental Language Disorder (DLD) https://www.dldandme.org/all-articles/diagnosing-and-treating-developmental-language-disorder-dld (accessed on 26 April 2026)
	DLD assessment: Start here https://www.theinformedslp.com/review/DLD-Assessment-Start-here (accessed on 26 April 2026)
	Diagnosing bilectal children with SLI: Determination of identification accuracy (relevant for Greek Cypriot children) https://www.tandfonline.com/doi/abs/10.1080/02699206.2016.1182591 (accessed on 26 April 2026)
	If we don’t look, we won’t see: Measuring language development to inform literacy instruction https://journals.sagepub.com/doi/full/10.1177/2372732219839075 (accessed on 26 April 2026)
1g. Knowledge of relevant policies and laws on the rights of children with languages difficulties	Look for country-specific legislations (e.g., Cyprus) https://www.cylaw.org/nomoi/enop/non-ind/1999_1_113/full.html (accessed on 26 April 2026)
**Domain 2: Attitudes and Views about DLD**	
2a. Stereotypes around DLD	7 myths about DLD and the truths behind them https://www.moorhouseinstitute.com/7-myths-about-dld-and-the-truths-behind-them/(accessed on 26 April 2026)
	Myths about Developmental Language Disorder https://www.dldandme.org/all-articles/myths-about-developmental-language-disorder (accessed on 26 April 2026)
	Developmental language disorder and associated misconceptions: a multi-country perspective https://www.researchgate.net/publication/352744572_Developmental_language_disorder_and_associated_misconceptions_a_multi-country_perspective (accessed on 26 April 2026)
	“They don’t realise how hard he has to try every day”: The rewards and challenges of parenting a child with developmental language disorder https://pmc.ncbi.nlm.nih.gov/articles/PMC11839224/(accessed on 26 April 2026)
2b. Participation and inclusion for children with DLD	Belonging in school https://inclusion.mrc-cbu.cam.ac.uk/ (accessed on 26 April 2026)
	“So, I told him to look for friends!” Barriers and protecting factors that may facilitate inclusion for children with Language Disorder in everyday social settings: Cross-cultural qualitative interviews with parents https://www.sciencedirect.com/science/article/pii/S0891422221001128 (accessed on 26 April 2026)
	Developmental language disorders and special educational needs: consideration of inclusion in the Norwegian school context https://www.frontiersin.org/journals/education/articles/10.3389/feduc.2024.1436298/full (accessed on 26 April 2026)
2c. Tailoring classroom environments to better support children with DLD	Hidden in plain sight: A qualitative exploration of teachers and children’s perspectives on supporting developmental language disorder in school https://journals.sagepub.com/doi/full/10.3233/ACS-220010 (accessed on 26 April 2026)
	DLD friendly audit www.naplic.org.uk/resource/dld-friendly/ (accessed on 26 April 2026)
	Stakeholder perspectives on educational needs and supports for students with developmental language disorder https://onlinelibrary.wiley.com/doi/abs/10.1111/1460-6984.13134 (accessed on 26 April 2026)
**Domain 3: Roles in Supporting Children with DLD**	
3a. Teachers’ roles in early identification of DLD and prevention	Taking Steps in the Right Direction: Considerations for Implementing Universal Oral Language Screenings in the Schools https://pubs.asha.org/doi/10.1044/2025_LSHSS-25-00008 (accessed on 26 April 2026)
3b. Teachers’ roles in supporting children with DLD in the classroom	Useful resources for primary teachers https://speechandlanguage.org.uk/educators-and-professionals/dld-educational-support/for-primary-teachers/(accessed on 26 April 2026)
3c. Teachers’ roles in interprofessional collaboration	Universal Design for Learning: A Shared Language to Create a Culture of Collaboration and Leverage Interprofessional Practice https://pubs.asha.org/doi/abs/10.1044/2025_LSHSS-24-00142 (accessed on 26 April 2026)
	SLP-educator classroom collaboration: A review to inform reason-based practice https://journals.sagepub.com/doi/10.1177/2396941516680369 (accessed on 26 April 2026)
	Interprofessional Practice Between Speech-Language Pathologists and Classroom Teachers: A Mixed-Methods Systematic Review https://pubs.asha.org/doi/full/10.1044/2023_LSHSS-22-00168 (accessed on 26 April 2026)

^1^ Note that hyperlinks may not be visibly marked; however, clicking on the associated text will redirect to the referenced source. This list of resources is not exhaustive as there are many more high-quality resources available online. It is important for selected resources to reflect the linguistic, policy, and educational context of the country in which trainings are provided.
